# To appreciate the influence of contributed determinants on dental care utilization in the context of socio-economic inequalities

**DOI:** 10.1186/s12939-024-02220-5

**Published:** 2024-07-17

**Authors:** Aydin Joudi, Katayoun Sargeran, Hossein Hessari

**Affiliations:** 1 Departments of Community Oral Health, School of Dentistry, Tehran University of Medical Sciences, Tehran, Iran; 2 Research Center for Caries Prevention, Dentistry Research Institute. Department of Community Oral Health, School of Dentistry, Tehran University of Medical Sciences, Tehran, Iran

**Keywords:** Dental care utilization, Inequality, Determinants, Concentration Index, Adults

## Abstract

**Background:**

To appreciate dental care utilization in ‌the context of socio-economic inequalities, it is imperative to identify sources of inequalities and evaluate the extent to which dental care utilization is still related to socio-economic status. This study aimed to quantify the influence of contributed determinants on dental care utilization in the context of socio-economic inequalities amongst adults residing in Tehran metropolis**.**

**Methods:**

In this cross-sectional community-based study, a stratified random sample of 1,510 subjects aged over 18 years was investigated by the zero-inflated Poisson analysis to measure the effect of determinants on utilization of dental care, and concentration index as well as the decomposition approach to identify the contributions of deterministic variables to the socio-economic inequality. Data was obtained by employing a phone interview survey. Individuals who were not willing or able to answer the questions in the telephone interview due to hearing or neurological problems did not participate in the interview. Dental care utilization was measured using the number of dental appointments.

**Results:**

Gender (male), oral health-related behaviors (such as brushing and dental flossing), experience of toothache, and concern about dental appearance were associated with an increased likelihood of utilizing dental care. Individuals who belonged to advanced age groups and lived alone significantly underutilized dental care. The concentration index equaling 0.05 (SE = 0.05) corroborates a pro-rich inequality. Decomposition analysis demonstrated the impact of oral health-related behaviors (i.e. dental brushing and use of dental flossing), concern about dental appearance, toothache, gender (male), insurance coverage of dental care, and smoking habit on the poor-rich gap in the dental care utilization.

**Conclusions:**

The influence of socio-economic inequalities on dental care utilization is discernable along the entire spectrum of socio-economic status. Individuals with lower socio-economic status experience more underutilization of dental care. Community subgroups, particularly the more deprived bracket, require consideration from key stakeholders, including policymakers and health professionals for the enhancement of dental care utilization as revealed by underlying determinants.

## Background

Individuals should access and utilize dental care if needed regardless of their socio-economic privileges. Contextual and socio-economic disparities make unequal access to dental care, and consequently, have a strong influence on individuals’ utilization of dental care and oral health status [1]. Inequality in dental care utilization is a major public health concern, and a pro-rich inequality in dental care utilization was identified in both developed and developing states [1–3].

Inequalities in dental care utilization lead to poorer oral health, particularly, in subjects with low socio-economic status (SES) [4]. The World Dental Federation (FDI) Vision 2030 favors the enhancement of oral health status and the decreasing inequalities in this context within the next decade. Likewise, globally equal access to dental care and coverage of universal care in promoting oral health status was identified as a priority by the FDI [5].

Dental care has been neglected for a long time, with health systems primarily focusing on basic healthcare services and high costs with limited insurance coverage make specialized dental care unaffordable [6]. Fortunately, the “Universal Health Coverage for Oral Health by 2030” emphasized oral health status. Several states initiated oral health promotion interventions or plans to implement them in the future [3]. These initiatives might ameliorate the accessibility to dental care services. This may lead to a decrease in inequality in dental care utilization.

The Health system in Iran mostly excludes dental care from its national health insurance coverage. Oral health departments in the primary health care (PHC) network provide dental care to the target groups (pregnant women, under 6 years subjects, and subjects aged between 6–12 years) in two levels. The first level includes oral hygiene education and preventive care (such as fluoride therapy and fissure sealant) and the second level of care mainly includes simple restoration, scaling, and primary root canal treatment such as pulpotomy [7]. Based on the report of the World Health Organization (WHO), among the Eastern Mediterranean countries, the out-of-pocket expenditure paid by individuals for dental care expenditures in Iran is between 51% and 90%, which ranks first grade [8].

The majority of Iranians pay for dental care through out-of-pocket expenditure or self-funded complementary health insurance with dental coverage, constituting 80% of all dental expenditures in the state. Insurance coverage influences the probability and rate of individuals’ dentist visits [9].

Indeed, individuals without insurance coverage, in particular, those with low SES experience more barriers in utilizing dental care. Thereby, these individuals potentially are more vulnerable to underutilization of dental care and consequently suffer from worse oral status. An investigation conducted within the population of Iran in 2021 stated that ‘Inequalities in dental care expenditure were in favor of individuals with higher education attainment level and economic status (i.e. high SES) [10]. Consistently, another study revealed a pro-rich concentration of dental care utilization. Likewise, income, provinces of residence, and education attainment level are addressed as the main indicators contributing to dental care utilization [2].

Urban Health Equity Assessment and Response Tool survey is one of the investigations conducted to identify health disparities in the metropolis of Tehran, the capital, and, the area with the most diverse population in the state [11]. However, large-scale surveys like this did not include dental care utilization measures. Hence, despite the crucial importance of socio-economic inequalities in dental care utilization, there is not currently much research investigating the impact of social determinants on inequalities in dental care utilization amongst adults residing in Tehran metropolis. Therefore, this study aimed to assess the contributed determinants of dental care utilization in ‌the context of socio-economic inequalities amongst adults residing in Tehran metropolis using the zero-inflated Poisson analysis and concentration index decomposition approach.

## Methods

### Study design and participants

This cross-sectional community-based study was conducted among individuals aged over 18 years residing in the Tehran metropolis. Participants were surveyed by the phone-based method between January 2023 and June 2023. In this study, participants’ information is securely held and will not be divulged anywhere. Ethical approval for this study was confirmed by the Ethics Committee of Health Sciences at Tehran University of Medical Sciences (IR.TUMS.DENTISTRY.REC.1400.182).

### Eligibility criteria

In this study, individuals who were not willing or able to answer the questions in the telephone interview due to (1) disabilities, (2) neurological impairment, dementia, and intellectual handicap, and (3) too ill/hard of hearing did not investigate.

### Measurements

The Structured questionnaire prepared for the data collection tool was anonymized so respondents could not be identified. The questionnaire was designed in three steps: In the first step, the content domains of the questionnaire were identified based on the Anderson model. In the following, the questions, per identified domains were designed using the literature, comments of the community oral health experts as well as authors’ personal opinions, and were made up of three sections:

**Section 1** included four questions to collect socio-demographic data: respondents self-reported age (18–34, 35–44, 45–64, 65 +), gender (Female/Male), educational attainment level (Illiterate/ primary and high school, High school diploma, and University level), living arrangement (Alone and Not alone), and dental insurance coverage (Yes, No).

The individuals’ socio-economic status was determined by the living area of their apartment/house in square meters per person (m^2^/p). This proxy measure was used because previous investigations among Tehran population demonstrated that living area in square meters per person is a reliable measure of individuals’ socio-economic status. The population of Tehran usually have few occupations and underestimation of self-reported income makes the income information unreliable [12].

**Section 2** consisted of questions to collect personal oral health-related behavior and knowledge: The frequency of toothbrushing (≥ Twice per day, Once a day, Less than once per day), daily use of dental floss (Yes, No), and smoking status (Active smoker, Ex-smoker, and Non-smoker).

**Section 3** contained four questions: three questions to evaluate subjective oral problems. The questions about subjective oral problems were: “Have you ever had pain in your mouth (such as a toothache) within the past 12 months?” (Yes/No), and “Have you usually experienced dryness in your mouth?” (Yes/No). “Have you ever had a concern about your dental appearance within the past 12 months?” (Yes/No). In order to evaluate the subjects’ oral health conditions, the question “How would you describe your oral health status?” was asked.

Participants declared their utilization of dental care services by answering the question ‘‘How many times did you visit the dentist within the last 12 months?’’.

The explanatory variables were selected based on the conceptual framework of the Andersen Model for the utilization of health care and previous literature on dental care utilization. The Andersen Model is a framework intended to explain contextually and individually contributed determinants including predisposing characteristics (gender, age, frequency of toothbrushing, daily use of dental floss, smoking status), enabling resources (educational attainment level, living arrangement, socio-economic status, dental insurance coverage), and needs factors (self-assessed oral health status, toothache within the past 12 months, dental appearance, dryness in the mouth) associated with healthcare outcomes such as dental care utilization [13].

### Sample size and sampling procedures

Based on the prevalence of dental care utilization (56%) obtained from the literature [14], we estimated the sample size with *n* = (z)^2 ^p ( 1 – p) / d^2^, which Z = the standard normal deviate (usually set at 1.96 which corresponds to the 95% confidence level), *p* = prevalence of dental care utilization, d = 0.0353, *n* = the desired sample size [15]. The minimum sample size was calculated at 759 subjects. The design effect was considered equal to two, and the final sample size required was equal to a population of almost 1,510 subjects. Proportionate stratified random sampling was used to ensure that the subjects were sampled from each 22 districts (strata) of the metropolis.

Phone-based surveys are considered a valuable and low-cost alternative to face-to-face interviewing or the use of mailed or group-administered questionnaires, especially in a broad geographical area data collection like a metropolis.

Twenty experienced and calibrated interviewers conducted the phone interviews. Initially, two monitored interviews were conducted. In two monitored interviews, feedback was provided to the interviewers, and questions were answered. During the data collection, the authors were presented among the interviewers and supervised the data collection process. The interviews were audio recorded and randomly 10% of them were reassessed by the authors. A random number generator was used to generate 75 numbers for each district employing known prefixes assigned to telecommunication services providers, and randomly select phone numbers. The calls were scheduled at different times of the day, the first call would be in the morning, and the other one during non-working hours if the first time, there was no answer.

Phone numbers were called and the subjects’ answering was queried regarding his/her eligibility criterion for this investigation. If the respondent was not eligible or willing to participate in the interview, he/she was asked if another member of the household was eligible and available. Sampling continued by interviewing that subject. Data collection continued until quotas for each district were reached. All participants declared verbal informed consent containing the participants’ identities and information before starting the interview. Verbal consent contained the components of informed consent, consisting of the aim of the study, the time required for the interview, the types of questions that interviews would ask, the benefits of this survey, and finally contact information for concerns and/or potential questions. The reportage of this study was in agreement with the STROBE statement: Strengthening the Reporting of Observational Studies in Epidemiology.

### Statistical analysis

To assess the content and face validity of the questionnaire, the opinions of five experts and faculties of community oral health were obtained on the accuracy, completeness and relevance, and the appropriate position of the words. Moreover, the questionnaire was evaluated with a sample of 20 lay cases from the target community. Moreover, the questionnaire was assessed by twenty experts and university professors in community oral health from different dental schools and they were asked to rate the necessity, relevance, simplicity, and clarity of each question [16]. To evaluate the reliability of the questionnaire, a pilot test was conducted. To examine the internal consistency, a total of 30 subjects aged over 18 years, were selected among residents of two metropolis districts and interviewed once more at the two-week interval.

The CVI (Content Validity index) and CVR (Content Validity Ratio) were calculated for content validity and all the items achieved the minimal acceptable level [16]. Spearman’s rank correlation coefficient was 0.65 (*P*-value = 0.01). The Cronbach’s alpha value of the questionnaire was acceptable at 0.81. Modifications were subsequently made to the protocol before initiation of the data collection procedure.

Not all information requested was provided by participants and the source of missing data was from incomplete responses. However, in all cases, the number of missing values was less than 5%. Hence, it was decided to use EM (Expectation–Maximization) imputation for the data that was missing. EM is an iterative maximum likelihood approach in a repeated cycle of mean and covariance calculation carried out by data imputation. This imputation continues until gaining a stable set of estimated missing values. Compared to means substitution, list-wise deletion, or pair-wise deletion, the EM estimation approach is preferable [17]. All explanatory variables were examined in a univariate analysis.

The zero-inflated Poisson (ZIP) regression was employed to obtain the incidence rate ratio of the outcome variable (utilization rate of dental care within the past 12 months) with predisposing, enabling, and needs factors. The ZIP regression is a model for analyzing count data with excess zeros. Probability p the only possible observation was assumed as 0, and the probability of a Poisson (λ) random variable, 1 – p is observed. This regression includes two separate models: a logit (binary) model for the ‘certain zero’ cases predicting whether or not an individual residing in the Tehran metropolis would have utilization of dental care and a Poisson model estimating the counts for the residents’ dental utilization who are not “certain zeros” [18]. To compare the ZIP model with the standard Poisson, a Vuong test was used. The overall fit of the predictors was ascertained by the Wald test.

Prior to the regression analyses, multicollinearity was assessed by using the variance inflation factor (VIF) test. The VIF as well as the tolerance values indicated no problems with multicollinearity as all values for VIF were 1.36 (VIF < 2.5) and the mean value of VIF was 1.36 [19, 20].

## Measuring socio-economic inequalities in dental care utilization

To assess socio-economic inequality in dental care utilization, we used the concentration index (CI) and the concentration curve. The concentration curve plots the cumulative share of dental care utilization (y-axis) against the cumulative proportion of the participants (x-axis) ranging from the 1st (the most deprived bracket) to the 5th (the least deprived bracket). A 45-degree line running from the bottom left-hand corner to the top right-hand corner (the equality line) determines the fair distribution of dental care utilization. The CI is described as twice the area between the concentration curve and the equality line and ranges from –1 to + 1. The positive CI demonstrates pro-rich inequality (i.e. the Concentration curve was below the equality line) and vice versa.

## Decomposing inequality in dental care utilization

Decomposition analysis was conducted to identify the contribution of explanatory determinants to inequality in dental care utilization. Each contribution shows how much variation in dental care utilization can explain the observed association between SES and dental care utilization. A contributed determinant with positive value contributes to more inequality in dental care utilization in favor of advanced SES brackets, and vice versa. Hence, inequality in dental care utilization increased by a positive contribution rate, and vice versa.

A zero-inflated Poisson model was employed for the decomposition of the CI and determinants that contributed to the inequality of dental care utilization [21].

1 $$yi=G(\alpha +{\sum }_{i}\left(\left(\beta i\right)xi\right))+{\varepsilon }_{i}$$

where G utilizes a particular form for the zero-inflated Poisson model. The concentration index (CI) for y, can be obtained as follows:

2 $$\text{CI}= {\sum }_{\text{k}}\left(\frac{\left({\upbeta \upkappa }\right)\stackrel{-}{\text{x}}\upkappa }{\upmu }\right)\text{C}\upkappa +\frac{\text{GC}\varepsilon }{\upmu }$$

 

where β_k_ is the regression coefficient, x̄_k_ is the mean of the variable k, µ is the mean of the interest determinants, and C_k_ is the C of the variable k. The second component of the formula, $\frac{\text{GC}\varepsilon }{\upmu }$, is the generalized C for the residual term ε [22].

C is reduced to the first component of Eq. and can be calculated by: 

3 $$\text{C }={\sum }_{\text{k}}(\frac{\left({\upbeta \upkappa }\right)\stackrel{-}{\text{x}}\upkappa }{\upmu })\text{C}\upkappa$$

In the above Eq., the value of $\frac{\left({\upbeta \upkappa }\right)\stackrel{-}{\text{x}}\upkappa }{\upmu }$ is described as the elasticity of the k indicator.

C for each indicator was estimated by the covariance formula of C, suggested by Wagstaff et al. [23]: 

4 $$\text{C }=\frac{2\text{cov}(\text{x},\text{ h})}{\upmu }$$

where cov(x, h) is the covariance between the dental care utilization and the SES rank of each explanatory variable. Consequently, the mean and the coefficient were estimated using the zero-inflated Poisson regression model, elasticity was obtained for decomposing the CI, and C_k_ was calculated for each explanatory determinant using the IV Eq. The contribution of each explanatory variable was estimated using multiplying the elasticity by 4.C_k_. Sensitivity analyses were employed to evaluate the robustness of the decomposition analyses.

Post-stratification weights were computed to adjust for differences in the age-by-gender between the sample and the population of Tehran. The statistical analyses were carried out by Stata 16 SE (Stata-Corp., College Station, TX). *P*-values < 0.05 were considered statistically significant.

## Results

Descriptive statistics of the study sample were presented in Table 1 presents. The response rate to our telephone interview was 52.4%. The total number of call attempts was 11,691, and 2,582 subjects refused to participate. The participants included 1,510 subjects with a mean age of 46.21 (SD = 18.43). Most of the participants (45.8%) were in the 18–34 age group and lived not alone (87.5%). Only 39.9% of the participants had an academic degree (Table 1).

**Table 1:** Socio-demographic characteristics of subjects aged over eighteen years, in Tehran metropolis, in 2022 (*N* = 1,510)

**Explanatory variables**		**n (%)** (Weighted)
***Predisposing characteristics and oral health-related behaviours***		
**Gender**	Male	753(49.9)
	Female	757(50.1)
**Age group**	18–34	691(45.8)
	35–44	273(18.1)
	45–64	394(26.1)
	+ 65	151(10.0)
**Smoking status**	Non-smoker	1310(86.8)
	Active smoker	154(10.2)
	Ex-smoker	46(3.0)
**Frequency of toothbrushing**	> Twice a day	440(29.2)
	Once a day	789(52.2)
	Less than Once a day	281(18.6)
**Daily use of dental floss**	Yes	426(28.2)
	No	1084(71.8)
***Enabling factors***		
**Educational attainment level**	Illiterate	90(5.9)
	Primary and high school	340(22.6)
	High school diploma	479(31.7)
	University	602(38.9)
**Living arrangement**	Alone	189(12.5)
	With others	1321(87.5)
**Dental insurance coverage**	Yes	411 (27.2)
	No	1099(72.8)
**Socio-economic status**	**Mean (SD)**	34.60 ± 24.82
***Oral health needs***		
**Self-assessed oral health status**	Excellent	126(8.4)
	Very Good /Good	660(40.1)
	Fair	390(25.8)
	Poor /Very poor	387(25.7)
**Toothache within the past 12 months**	Yes	692(45.8)
	No	818(54.2)
**Dental appearance**	Yes	425(28.2)
	No	1085(71.8)
**Dryness in the mouth**	Yes	169 (11.2)
	No	1341 (88.8)
***Outcome determinant***		
**Utilisation of dental care within the past 12 months**		**Median (IQR) = **1.0(2.0)

*SD* Standard Deviation, *IQR* Interquartile Range

To identify the influence of the predisposing, enabling, and needs characteristics on the utilization of dental care, all of the determinants were considered in the zero-inflated Poisson model. Upon controlling for all determinants in the model, statistically significant associations were found for the age groups. Individuals who belonged to advanced age groups significantly underutilized dental care compared to those who were in the 18–34 age group within the last 12 months. This decrease was sharp in the elder group.

Males revealed an increased likelihood of utilizing dental care (IRR = 1.32, *P*-value < 0.001). The likelihood of visiting a dentist (i.e., once or more per year) was decreased by a factor of 0.57 for people living alone (*P*-value < 0.001). Oral health-related behaviors (i.e. brushing and dental flossing) were associated with more frequent utilization of dental care. However, smoking habit showed an inverse significant association with dental care utilization.

In the bracket of needs factors, it appears that there are no statistically significant associations between the likelihood of dental care utilization and self-assessed oral health status. The likelihood of visiting the dentist more frequently was increased for people who had toothache within the last 12 months compared to those who did not (IRR = 1.12, *P*-value < 0.029). Having concern about dental appearance was associated with the likelihood of increased dental care utilization (IRR = 1.41, *P*-value < 0.001). (Table 2). It was found that subjects with usual dryness feeling in the mouth were more likely to utilize dental care compared to subjects who did not have it. However, this feeling was not statistically significant.

**Table 2:** Zero-inflated Poisson regression analysis between utilisation of dental care (dependent variable) and explanatory variables amongst subjects aged over eighteen years, in the Tehran metropolis, in 2022 (*N* = 1,510)

**Explanatory variables**		**Univariate analysis**		**Multivariable analysis** **(final model)**		
		**IRR (95% CI)**	*P-*value	**IRR (95% CI)**	*P-*value	
***Predisposing characteristics and oral health-related behaviours***						
**Gender** **(Female**^***Ref***^**)**	Male	**1.38 (1.26,1.52)**	< 0.001*	**1.32 (1.19,1.45)**	< 0.001*	
**Age group** **(18-34**^***Ref***^**)**	35–44	1.01 (0.90,1.14)	0.795	**0.83 (0.72,0.94)**	0.006*	
	45–64	0.91 (0.82,1.01)	0.074	**0.72 (0.64,0.81)**	< 0.001*	
	≥ 65	**0.22 (0.19,1.27)**	< 0.001*	**0.38 (0.30,0.48)**	< 0.001*	
**Frequency of toothbrushing (Less than once a day**^***Ref***^**)**	≥ Twice a day	**1.33 (1.22,1.46)**	< 0.001*	**1.56 (1.33,1.82)**	< 0.001*	
	once a day	**0.83 (0.76,0.91)**	< 0.001*	**1.18 (1.01,1.38)**	0.033*	
**Daily use of dental floss (No**^***Ref***^**)**	Yes	**1.23 (1.12,1.35)**	< 0.001*	**1.20 (1.09,1.32)**	< 0.001*	
**Smoking status** **(Non-smoker**^***Ref***^**)**	Ex-smoker	**1.28(1.00**^**+**^**,1.32)**	0.035*	**1.32 (1.05,1.66)**	0.017*	
	Active smoker	1.14 (0.99,1.31)	0.071	**1.20 (1.04,1.38)**	0.013*	
***Enabling factors***						
**Educational attainment level** **(Illibrate/Primary and high schoo**l^***Ref***^**)**	High school diploma	1.06 (0.97,1.17)	0.197	1.01 (0.90,1.14)	0.812	
	University	0.92 (0.84,1.01)	0.090	0.93 (0.82,1.04)	0.202	
**Living arrangement** **(With others**^***Ref***^***)***	Alone	**0.64 (0.54,0.76)**	< 0.001*	**0.57 (0.48,0.68)**	< 0.001*	
**Socio-economic status**		**1.01 (1.00,1.02)**	0.022*	1.01 (1.00,1.03)	0.256	
**Dental insurance coverage (No**^***Ref***^**)**	Yes	1.08(0.98,1.19)	0.124	1.05 (0.96,1.16)	0.292	
***Oral health needs***						
**Self-assessed oral health status** **(Poor/Very poor**^***Ref***^**)**	Excellent	0.96 (0.87,1.06)	0.424	1.06 (0.87,1.28)	0.570	
	Very Good/Good	**0.82 (0.75,0.92)**	< 0.001*	0.94 (0.82,1.07)	0.356	
	Fair	1.04 (0.89,1.22)	0.612	0.95 (0.83,1.08)	0.435	
**Toothache within the past 12 months (No**^***Ref***^**)**	Yes	**1.38 (1.25,1.51)**	< 0.001*	**1.12 (1.01,1.25)**	< 0.029*	
**Dental appearance** **(No**^***Ref***^**)**	Yes	**1.60 (1.46,1.75)**	< 0.001*	**1.41 (1.26,2.56)**	< 0.001*	
**Dryness in the mouth** **(No**^***Ref***^**)**	Yes	**1.27 (1.11,1.45)**	< 0.001*	1.09 (0.95,1.25)	0.236	
**Explanatory variables**		**Univariate analysis**		**Multivariable analysis** **(final model)**		
		**exp(b) (95% CI)**	*P-*value	**exp(b)(95% CI)**	*P-*value	
***Poisson* = *0***						
**Gender** **(Male**^***Ref***^**)**	Female	0.06 (-0.18,0.30)	0.608	**-1.41(-1.83, -0.99)**		<0.001*
**Age group (≥ 65**^***Ref***^**)**	18–34	**-0.93 (-1.18,-0.68)**	< 0.001*	**-1.11 (-1.59, -0.63)**		<0.001*
	35–44	**-0.37 (-0.68,-0.05)**	0.022*	**-1.25 (-1.71, -0.79)**		<0.001*
	45–64	**-0.47 (-0.75,-0.20)**	0.001*	**1.41 (1.-0,1.83)**	< 0.001*	
**Educational attainment level** **(Illibrate/Primary and high school**^***Ref***^**)**	High school diploma	-0.11 (-0.40,014)	0.336	-0.02 (-0.38,0.33)	0.897	
	University	0.11 (-0.12,0.34)	0.346	-0.11 (-0.23,0.46)	0.522	
**Toothache within the past 12 months (Yes**^***Ref***^**)**	No	**0.97 (0.74,1.20)**	< 0.001*	0.74 (0.45,1.02)	< 0.001*	
**Dental insurance coverage (Yes**^***Ref***^**)**	No	**0.61 (0.38,0.84)**	< 0.001*	**0.58(0.25,0.91)**	< 0.001*	

*IRR* Incidence Rate Ratio

*P-*value < 0.05* (in **bold**), Wald Χ^2^ (20) = 349.23, *P-*value** < 0.001**, **Vuong test**: 6.40, *P-*value < 0.001, Bayesian information criterion (BIC) difference = 414.626. Akaike information criterion (AIC) difference = 462.505

The zero-inflated Poisson regression model was significantly better than the Poisson model (z = 6.40, *P*-value < 0.001), according to the result of the Vuong test, thus the zero-inflated model was employed for statistical analysis.

**Fig. 1:**
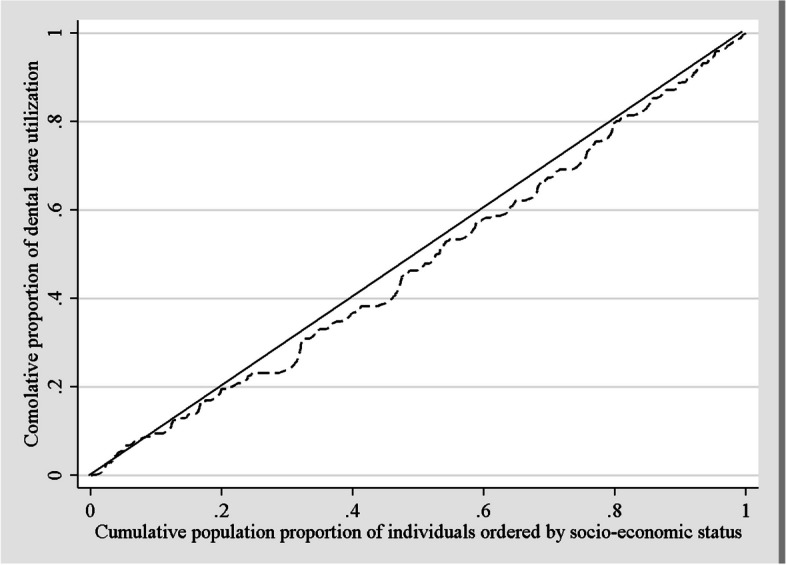
The concentration curve of dental care utilization within the past 12 months by socio-economic status amongst adults, in Tehran metropolis, in 2022 (*N* = 1,510)

Study results indicated that the concentration index of dental care utilization was 0.05 (SE = 0.05) among adults in the metropolis of Tehran. The concentration index had a positive value, which was aligned with the Concentration curve. Figure 1 shows a socio-economic gradient of dental care utilization in the metropolis of Tehran. The Concentration curve was below the equality line. As illustrated in the curve, within the population of Tehran, a pro-rich utilization of dental care was detected (Fig. 1).

The decomposition results of inequality in dental care utilization by socio-economic status were presented in Table 3. A major increasing contributor to the inequality of dental care utilization was oral health-related behaviors (i.e. dental brushing and use of dental flossing), concern about dental appearance, experience of toothache within the past 12 months, and gender (male). The other strong contributors were the insurance coverage of dental care and smoking habits with contribution percentages of 13.95 and 16.79%, respectively. The likelihood of dental appointments was increased for subjects who experienced toothache, and this led to more inequality in dental care utilization with a relative contribution percentage of 23.96%. Twice a day toothbrushing was concentrated in the more advantaged bracket. Moreover, living alone, age increasing, and better self-assessed oral health status inversely contributed to this inequality.

**Table 3:** Decomposition of Inequality in utilisation of dental care by economic status amongst subjects aged over eighteen years, in Tehran metropolis, in 2022 (***N*** = 1,500)

Explanatory variables		CI	Elasticises	Con	Percentage con
***Predisposing characteristics and oral health-related behaviors***					
**Gender (Female**^***Ref***^**)**	Male	0.0835	0.1030	0.0344	41.21
**Age group (18-34**^***Ref***^**)**	35–44	-0.1480	-0.0341	0.0202	-13.63
	45–64	-0.0362	-0.0774	0.0112	-30.96
	+ 65	-0.0619	-0.1252	0.0310	-50.08
**Smoking status** **(Non-smoker**^***Ref***^**)**	Ex-smoker	0.1975	0.0134	0.0106	5.37
	Active smoker	-0.3160	0.0285	-0.0361	11.42
**Frequency of toothbrushing** **(Less than Once a day**^***Ref***^**)**	> Twice a day	0.2072	0.1223	0.1013	48.90
	Once a day	-0.0444	0.0822	-0.0146	32.86
**Daily use of dental floss** **(No**^***Ref***^**)**	Yes	0.1004	0.0550	0.0221	22.02
***Enabling factors***					
**Educational attainment level** **(Illiterate** /**Primary and High schoo**l^***Ref***^**)**	High school diploma	0.1192	-0.0175	-0.0084	-7.01
	University	0.0416	-0.0452	-0.0075	-18.06
**Living arrangement** **(With others**^***Ref***^***)***	Alone	0.0808	-0.0579	-0.0187	-23.16
**Dental insurance coverage (No**^***Ref***^**)**	Yes	0.0420	0.0349	0.0059	13.95
**Socio-economic status**		0.8342	-0.0078	-0.0262	-3.14
***Oral health needs***					
**Self-assessed oral health status** **(Poor/ Very poor**^***Ref***^**)**	Excellent	0.2524	-0.0050	0.0050	1.98
	Very Good /Good	0.0216	-0.0151	-0.0013	-6.05
	Fair	0.0185	0.0050	0.0004	2.01
**Toothache within the past 12 months (No**^***Ref***^**)**	Yes	-0.0344	0.0599	-0.0082	23.96
**Dental appearance** **(No**^***Ref***^**)**	Yes	-0.0754	0.1112	-0.0335	44.49
**Dryness in the mouth** **(No**^***Ref***^**)**	Yes	-0.0582	0.0028	-0.0007	1.14
***Residual (Unexplained) term***				0.0025	2.77

Elasticity is the effect of an explanatory determinant on utilisation of dental care concerning the mean of that determinant; CI is the concentration indices of each determinant; Con. is the contributions of an explanatory determinant to the overall inequality in utilisation of dental care; %Con. is the percentage contribution; Residual term is an unexplained component

## Discussion

This study the contributed determinants of dental care utilization in ‌the context of socio-economic inequalities using the zero-inflated Poisson analysis and concentration index decomposition approach amongst adults residing in Tehran metropolis. The findings of this study revealed a pro-rich inequality in dental care utilization in Tehran. Moreover, the decomposition analysis revealed that gender, self-assessed oral health status, smoking status, frequency of toothbrushing, daily use of dental floss, living arrangement, dental insurance coverage, the experience of toothache within the last 12 months, and concern about dental appearance strongly contributed to this inequality. Indeed, socio-economically disadvantaged subjects in both developed and developing societies tend to underutilize dental care. Consistent with our study, pro-rich inequality in dental care utilization among adults was acknowledged in studies conducted in developed and developing societies, such as European countries [24], Thailand [25], and China [3].

Enabling determinants (i.e. dental insurance coverage, and living arrangements) strongly contributed to the inequality in dental care utilization in favor of more privileged individuals. In other words, high out-of-pocket expenditure and reliance on private insurance generate a pro-rich disparity in the utilization of dental care. Individuals with dental insurance coverage have fewer hurdles before obtaining dental care. However, dental insurance coverage is provided mostly by private insurance organizations and is concentrated among the better-off. Universal coverage of dental insurance against the cost of prevention and treatment of caries and periodontal diseases can decrease inequality in dental care utilization and promote oral health. Hereupon, expanding the universal coverage of dental insurance ameliorates the affordability of dental care and decreases pro-rich inequality.

Living not alone increases the likelihood of dental care utilization, suggesting that social support influences the utilization of dental care. When there are individuals who reside in the household, the opportunity to take advantage of the available social supports (informational, emotional, transportation, etc.) increases. This leads to enable individuals, especially older adults to seek and obtain dental care more easily. Indeed, living alone and social isolation have been identified as a risk indicator for poor mental and physical health and have been associated with reduced social networks, restricted economic resources, and changes in family structure, particularly in older adults.

The experience of toothache was one of the main contributors to this inequality. As Lee and Lupi-Pegurier [26] showed, individuals who live in areas with a high density of dental care practitioners are more prone to use dental care. Residents in deprived neighborhoods have scanty financial and physical access to dental care providers. Deprived residents prefer to seek dental care only in severe oral conditions. Consequently, instantaneously and sufficiently met of these persons’ dental needs do not occur, due to restricted access to dental care services. This leads to an increase in the pro-rich inequality in dental care utilization.

Daily use of dental floss was positively associated with dental care utilization among the adults of Tehran. As confirmed by the literature, dental flossers experience less dental caries compared to non-flossers and have improved oral health [27]. On the other hand, subjects who assessed their oral health as poor/very poor and individuals with severe tooth loss significantly had less utilization of dental care than those with good self-rated oral health conditions [28, 29]. In other words, it appears that dental care utilization and oral health-related behaviors like flossing do not seem to be a priority in individuals with poor oral health. This leads to increased inequality in dental care utilization.

The results of this study revealed that more frequent toothbrushing positively contributed to the pro-rich inequality in dental care utilization among adults, which is confirmed by the findings of a systematic review in 2021 that revealed in frequent toothbrushing was associated with a higher risk for caries and consequently a higher need for dental treatments [30]. This suggests that proposing community-based policies aimed at enhancing oral health-related awareness about the crucial impact of good oral hygiene may be a more desirable solution.

Based on the findings of this study, smoking increased the likelihood of dental care utilization, suggesting that smokers are more susceptible to dental problems such as experience of toothache dentine, pains in the orofacial area, and teeth hypersensitivity. After the appearance of oral problems, the tendency to utilize dental care increases among smokers [31]. In other words, as Ide et al. stated smoking could be the cause of dental care utilization. Consist of this finding, a cohort investigation in Finland, the likelihood of dental appointments and dental care utilization enhanced with increased dental care expenditure among smokers [32]. The expenditure associated with smoking includes not only direct costs but indirect costs such as time required for physical access and dental treatment with working time lost as well [33]. These expenditures increased the pro-rich inequality among the residents of the metropolis.

Ultimately, the findings of this study are consistent with the results of the previous studies demonstrating that inequalities in the utilization of dental care are not merely driven by socio-economic determinants [30, 34]. Preferences related to living arrangements**,** oral health-related behaviors, the availability of dental care, psychosocial well-being, and morbidity status (such as a recent experience of toothache) have a substantial influence on dental care utilized by individuals.

## This study had limitations mentioned as follows

First, this study cannot establish causality between dental care utilization and the explanatory determinants, due to its cross-sectional design. Additionally, the cross-sectional design did not permit us to evaluate the influence of public policies on decreasing the inequalities in dental care services utilized by adults in the metropolis of Tehran.

Second, as the data were collected through individuals’ self-reports, this may have led to recall bias. Although there were some limitations, this study provides evidence of an inequality in dental care utilization among residents of Tehran metropolis aged over eighteen years. Hence, based on the findings of this study, future research should focus on the indicators created unfairly, such as unfair access to dentists/dental specialists, health insurance, and other health system characteristics.

Third, as individuals with too ill/hard hearing and neurological problems were not investigated in this interview, and this group is susceptible to social, economic, and inequality issues, it's crucial to specify inequality in dental care utilization and the influenced determinants on dental care utilization in this group.

## Conclusions

This study substantiated the presence of a pro-rich inequality in the utilization of dental care among individuals residing in the metropolis of Tehran aged over eighteen years. Gender, oral health-related behaviors (i.e. brushing and dental flossing), experience of toothache within the last 12 months, and having concern about dental appearance revealed an increased likelihood of utilizing dental care. Individuals belong to advanced age groups, live alone, significantly underutilized dental care. This decrease was sharp in the elder group. Smoking habits showed an inverse significant association with dental care utilization.

A major increasing contributor to the inequality of dental care utilization was oral health-related behaviors (i.e. dental brushing and use of dental flossing), concern about dental appearance, toothache within the past 12 months, and gender. The other strong contributors were the insurance coverage of dental care and the smoking habit. Moreover, living alone, age increasing, and better self-assessed oral health status inversely contributed to inequality.

To create equitable dental care utilization, social and multidisciplinary policies aiming to enhance oral health-related awareness of the main contributed determinants should be on the political agenda. Fairness-oriented health provision and financial systems should be established and strengthened. This study can apprise stakeholders and policymakers concerning decision-making around the allocation of enabler resources to subjects and communities with more oral health-related needs.

## Data Availability

To protect the privacy of the individuals, the dataset that supports the results of this paper is not publicly available. The data employed and analyzed in this study are available from the corresponding author upon reasonable request.

## Abbreviations

UHC: Universal Health Coverage
PHC: Primary Heath Care
SES: Socio-economic Stats
SD: Standard Deviation
IQR: Interquartile Range
CI: Concentration Index
CVI: Content Validity Index
CVR: Content Validity Ratio
CIs: Confidence Intervals
M: Mean value
IRR: Incidence Rate Ratio
Con: Contribution
TUMS: Tehran University of Medical Sciences
HIES: National Household Income and Expenditure Survey
